# Prevention and Control of COVID-19 Pandemic on International Cruise Ships: The Legal Controversies

**DOI:** 10.3390/healthcare9030281

**Published:** 2021-03-04

**Authors:** Xiaohan Zhang, Chao Wang

**Affiliations:** 1 Guanghua Law School, Zhejiang University, Hangzhou 310008, China; zhangxiaohan@zju.edu.cn; 2 Academy of International Strategy and Law, Zhejiang University, Hangzhou 310008, China

**Keywords:** COVID-19, infectious disease, international cruises, health policy and regulation, control strategies, international cooperation, global health governance

## Abstract

During the COVID-19 pandemic in 2020, a number of international cruise ships were infected, thereby resulting in serious public health and human rights problems. Multiple difficulties were encountered in the prevention and control of the coronavirus disease onboard ships, while rule-based international cooperation in this regard appeared inefficient and ineffective. By applying interdisciplinary methodologies, including empirical research of law, policy science, and health studies, this research reviewed the legal difficulties in the prevention and control of COVID-19 on international cruise ships and sought solutions from a policy-making and strategic perspective. We found that, apart from the inherent nature of cruise ships such as crowded semi-enclosed areas, shared sanitary facilities and limited medical resources, there are also nonnegligible legal reasons affecting the effectiveness of containment measures on board. In particular, there is ambiguity and even inconsistency of relevant international norms and domestic regulations, and some of the key rules are neither mandatory nor enforceable. We conclude by suggesting that rule-based international cooperation on this issue must be strengthened with respect to information sharing and management, a more effective supervisory mechanism, clarification of key rules over jurisdiction and distributions of obligations among the port states, flag states, nationality states, and cruise ship companies.

## 1. Introduction

International cruise tourism is the fastest growing sector of the travel industry since the early 1990s. Statistics show that in the past decade, cruising around the world has continued to boom with an average annual growth rate of 6.8 percent, and it was estimated that in 2020 the global ocean cruise industry would carry over 32 million passengers [1]. However, the sudden outbreak of COVID-19 at the end of 2019 deeply impacted this $150 billion industry as a number of international cruise ships were infected. The British-registered Diamond Princess cruise ship was the first one to have a major onboard outbreak, with over 700 people being infected, and the ship being quarantined at the Yokohama port of Japan on 4 February 2020, for nearly one month. By the end of May 2020, over 40 cruise ships had confirmed coronavirus cases. The last infected cruise ship with passengers onboard during the first wave of COVID-19, the German-based Artania, docked at its home port with its last passengers on 8 June 2020 [2]. During this period, many countries closed their borders and blocked international cruise ships from docking in order to prevent and control the pandemic. The United States Centers for Disease Control and Prevention (CDC), for instance, issued a No Sail Order effective on 13 March 2020 that suspended all cruise ship passenger operations [3]. As a consequence, thousands of passengers were quarantined on board for weeks before coming ashore, while seafarers were trapped at sea for an even longer time before being repatriated, resulting in “a humanitarian, safety and economic crisis” as described by the International Maritime Organization (IMO) [4].

Outbreaks of COVID-19 on international cruise ships attracted worldwide concern not only from stakeholders including cruise lines and national governments but also from researchers and the public. As a matter of fact, due to the inherent features of cruise ships such as the high population density, shared food supplies, and semi-enclosed living environments, the spread of infectious diseases occurs relatively easily on board [5]. There is scientific evidence in existing epidemiological studies suggesting that respiratory diseases, including influenza, legionnaires’ disease, avian influenza A(H7N9) and Middle East Respiratory Syndrome (MERS), are all among the most dangerous and high-risk viruses on cruise ships [6,7]. Once such infectious diseases break out on board, the viruses usually transmit rapidly and lead to public health emergencies that pose substantial challenges to the safety of ports and coastal states. During the COVID-19 pandemic, there is clear evidence that passengers aboard cruise ships played a role in spreading the coronavirus disease to a number of countries [8].

Both the prevention and the control of infectious diseases on cruise ships are relatively more complicated and problematic, especially for those international ships with passengers of different nationalities and docking ports located in different countries. Apart from the limited healthcare and medical conditions onboard, there are also difficulties with respect to the rule-based international cooperation and coordination of treatment measures. During the first wave of the COVID-19 pandemic, a number of international cruise ships were denied from docking or entering into the costal ports, as states applied different, changing, and sometimes even conflicting rules. The Holland America *MS Westerdam* cruise ship is a typical case in point. After departing from Hong Kong with 1455 passengers and 802 crew members on 1 February 2020, because of suspected coronavirus cases on board, the ship was denied entry not only by its destination port of Yokohama but also by other nearby ports in Japan, South Korea, Guam, Thailand, and the Philippines. On 13 February 2020, the ship was finally accepted by Sihanoukville Port in Cambodia, ending its two weeks’ helplessly drifting at sea. From a legal perspective, do these states have the right to deny entry? Which state is obligated to provide assistance in a public health emergency? If an infected ship is permitted to dock and disembark passengers, what measures can the port state take under its governing laws? How can it be ensured that all involved parties, including the flag state, the coastal state, and the ship operator/owner’s state, will cooperate effectively in the face of a global pandemic? All these issues are important for international cruise ships to prevent and control the on-going COVID-19 pandemic. However, they largely remain unclear and even unanswered. Existing literature and research mainly focus on textual interpretation of relevant legal provisions under normal circumstances, but without concurrently taking into account the unique features of international cruise ships. Much less is discussed under the on-going COVID-19 pandemic circumstance, which have brought upon unprecedented new challenges to the whole world.

Against this backdrop, this research article aims to analyze the international regulatory issues relating to the prevention and control of epidemics on international cruise ships, with a special focus on investigating those legal mechanisms from the perspective of strategies and policies of epidemic prevention and control. Section 2 introduces the collection of data, research materials, and methods of this research. Section 3 presents the research findings about the various legal difficulties of preventing and controlling COVID-19 on international cruise ships, and briefly summarizes the legal issues and conclusions. Section 4 further analyzes and discusses those regulatory issues in detail. Section 5 provides suggestions for addressing the aforementioned issues.

## 2. Materials and Methods

### 2.1. Research Data

Regarding the facts about outbreaks of the COVID-19 pandemic on international cruise ships, relevant information and data were collected mainly through the official websites of involved institutions, such as the International Maritime Organization (IMO), the International Cruise Line Association (ICLA), and other public sources such as Wikipedia and international news reports. The searching period for the factual data is set between January 2020 and December 2020.

For policies and strategies of COVID-19 prevention and control on cruise ships, research materials and data were collected mainly through relevant governmental departments, such as Japan’s Ministry of Health, Labour and Welfare, China’s National Health Committee and Maritime Safety Administration, and the United States Centers for Disease Control and Prevention (CDC) as well as its Vessel Sanitation Program (VSP). For academic analysis and discussions of these policies and strategies, research literature and references were obtained by searching databases such as PubMed, Medline, and Embase using keywords cruise ships, infectious disease, COVID-19, travel health, ship sanitation, and PHEIC.

For research questions on the governing laws and precedent cases, such as the interpretation and application of relevant provisions of the United Nations Convention on the Law of the Sea (UNCLOS), the WHO’s International Health Regulations (IHR), and the IMO’s Guidelines on Places of Refuge for Ships in Need of Assistance, references were acquired by searching Heinonline, Westlaw, LexisNexis and other professional legal databases.

### 2.2. Research Methods

Interdisciplinary methodologies including empirical research of law, policy science, and health studies were adopted. The comprehensive search and literature review of COVID-19 cases that are linked to cruise ships were conducted so as to provide a strong basis for empirical analysis and further discussions. Past public health incidents on international cruise ships during other pandemics such as SARS and MERS are also referred to. Based on findings of these facts, the actual effects of relevant rule-based mechanisms on tackling cruise ships’ public health emergencies are evaluated. These empirical studies will facilitate our understanding on the functioning of relevant legal regime and its impact on the formulation of pandemic control policies in the face of the COVID-19.

Doctrinal research, named “black letter” methodology, is fundamental to the study of legal issues. We will identify, describe, and critically analyze the text of relevant legal provisions contained in the UNCLOS, the IHR, and other relevant international conventions, as well as relevant domestic regulations of Japan, the United States, and other countries. The aim of textual analysis is to explore the original intention of legislation and identify ambiguities, inefficiencies, and even inconsistencies in relevant rules, based on which improvement suggestions and solutions are provided.

Case studies are also important for this research. We particularly focused on those representative cases such as the Diamond Princess cruise ship, which is considered a de facto epidemiological laboratory during the first wave of COVID-19 outbreaks in 2020. Lessons learned from this high-profile case are worth carefully studying in terms of strengthening rule-based international cooperation and improving health conditions on future cruise ships in similar pandemic situations. Major questions of these case studies include emerging regulatory issues encountered by various parties, whether and to what extent they are liable, whether their measures comply with relevant international and national norms and whether the right to health of people on board is sufficiently protected.

## 3. Results

Through data retrieval and analysis, we found that there was a COVID-19 pandemic outbreak on nearly 50 international cruise ships. A number of typical cases are listed in Table 1 below, which clearly indicates the complexity of this issue [9]. We also found that different cruise ships, ports, and coastal states adopted different measures to prevent and control the pandemic, which directly led to controversies. In particular, many ports were closed for travel restrictions and denied entry to international cruise ships and foreign nationals. Some countries such as Australia and the United States banned all foreign flagged ships from docking and directed them to leave, making no allowance for disembarkation. As a consequence of these controversial measures, thousands of passengers and crew members around the world were stranded on board and unable to be repatriated home.

The factors resulting in the difficulties of COVID-19 prevention and control on international cruise ships, apart from those inherent physical circumstances such as close living and working conditions and medical treatment restrictions on board, we found that the inadequacy and even failure of cooperation by involved parties were incredibly salient. The WHO has indicated that health on international cruise ships is a shared responsibility of all relevant stakeholders, involving equitable access to essential care and collective defense against transnational threats [10]. In terms of international cooperation when there is a Public Health Emergency of International Concern (PHEIC), ideally, all involved parties and states could make effort to cooperate with each other, whether for humanitarian purposes or to fulfill a specific obligation. During the first wave of the COVID-19 pandemic, we found that the fundamental international cooperation was weak. By taking cooperation under the United Nations framework as an example, although there are several specialized agencies such as the WHO, the ILO, and the IMO that are closely connected with the prevention and control of COVID-19 on international cruise ships, cooperation among them remains largely at the level of making joint declarations, with few joint actions. As a result, the functioning of these key international organizations appears inefficient and ineffective in the face of the COVID-19 pandemic.

We further found complicated legal motivations, as all measures and cooperation are rule-based. A large number and variety of laws and regulations, both national and international, are applicable to the issue of prevention and control of COVID-19 on international cruise ships. The UNCLOS, the foremost international agreement on law of the sea, for example, provides rules including the general obligation of maritime rescue and cooperation, jurisdiction of port states and flag states. Meanwhile, more substantial regulations on the management of ships and ports, entry and exit control, and health and quarantine requirements are contained and scattered in other international and national laws of the countries involved. In this regard, we discovered that there are certain ambiguity and even inconsistency with regard to the relevant international norms and the domestic regulations of individual states. Such a lack of regulatory harmony directly led to problems of conflicted jurisdictions, unbalanced liabilities, and an uncertainty of rescue obligations during the COVID-19 pandemic.

Moreover, some key rules are not enforceable or mandatory. For example, in order to cope with coastal states’ prohibition of environmentally threatening foreign ships from entering their ports, the IMO passed two resolutions to address the issue of places of refuge for ships in distress: the Guidelines on Places of Refuge for Ships in Need of Assistance [11] and the Maritime Assistance Services [12], which contain provisions for the coastal states to establish a ship refuge system. Nevertheless, these norms are soft law in nature and are not mandatory for implementation by any party. Under the PHEIC circumstances, in consideration of other factors such as self-safety, high risks and costs, environment protection and even geopolitics, most coastal states are understandably disinclined to accept infected ships entering their refuge areas. As a consequence, those distressed ships usually could not be timely and efficiently rescued. Similarly, we found that another key international organization, the WHO, only has limited legislative and enforcement power to regulate epidemic control measures on international cruise ships, while most of its IHR provisions serve more as guidelines for the national governments. The free pratique principle and restrictions on it is a case in point in terms of implementing the IHR regulations during the COVID-19 pandemic.

## 4. Discussion

### 4.1. Free Pratique, Rescue Obligation, and Refusal of Entry

#### 4.1.1. Application of the Free Pratique Principle

As a general principle, according to the IHR, a country shall grant ships the right of free pratique; that is, ships shall not be prevented from calling at any point of entry for public health reasons [13]. This is also in line with relevant UNCLOS provisions which require the coastal states to recognize the right of innocent passage for foreign ships [14]. Therefore, at the early stage of the COVID-19 outbreak, the WHO together with the IMO had called on all countries to respect the free pratique principle when noticing that several international cruise ships either experienced delayed port clearance or were denied entry to ports because of the coronavirus [15].

A limitation to the free pratique principle is that, when infection or contamination sources are found on board, a country may require disinfection, decontamination, disinsection or deratting, or other necessary measures that should be taken to prevent the spread of the infection or contamination [13]. The IHR also authorizes a country to take additional health measures pursuant to its national law, given that such measures are based on scientific principles, available scientific evidence of risk to human health, and the specific guidance or advice from the WHO [16]. Accordingly, a justified decision on whether there is a risk to human health requires actual and reliable research data; otherwise, the additional measures taken by an individual country will likely be challenged by other parties for lacking necessary evidence. This is the underlying reason why controversies arose when, during the COVID-19 pandemic, international cruise ships were denied entry into a number of costal states without justifications. For application of the “free pratique” principle, despite the insufficiencies of implementing relevant IHR provisions, whether a cruise ship might result in the spread of the coronavirus or bring other risks to the coastal states needs to be assessed with sufficient scientific methods. It is a scientific rather than a legal issue.

Though the UNCLOS allows costal states to deny a foreign ship’s right of innocent passage in their territorial waters and prohibit ships from entering for sanitary reasons [17], some ships were actually denied entry into ports without an evidence-based risk assessment (EBRA). The dilemma under the COVID-19 circumstance is that, on the one hand, the EBRA shall be based on actual and existing scientific data and requires professionals to use scientific methods to analyze the relevant data of the coronavirus; on the other hand, the coronavirus as a new disease has a certain degree of concealment in terms of its detection, infection, and transmission, especially at the early stage when not much was known. Therefore, the uncertainty of necessary scientific evidence would lead to hysteresis and insufficiency in applying those rules.

#### 4.1.2. Rescue Obligations of Coastal and Port States

From a legal perspective, when a pandemic outbreak occurs on board and an international cruise ship needs to be rescued, the first thing to ascertain is which country has an obligation to rescue. The UNCLOS stipulates two major types of maritime states: the coastal states and the port states [18]. According to its Article 98, every coastal state shall promote the establishment, operation and maintenance of an adequate and effective search and rescue service regarding safety at sea and where circumstances require cooperation with neighboring states for this purpose. Outbreak of COVID-19 on cruise ships would inevitably endanger passengers and crew members on board as well as the general safety at sea. In this sense, all costal states are under the general obligation of international law to rescue infected ships and people under their jurisdiction.

There are virtually two types of port states: one involves a cruise ship that has already entered the port, and the other involves a scheduled port of call without entering. In the latter case, according to relevant provisions of the IHR [19], if the port of call is an international sanitation port as accredited by the WHO, implying that it has certain sanitary facilities and the necessary capability to take such measures as quarantine on board or ashore, then its state should fulfill the relevant rescue obligations. In addition, the state of the ship’s home port shall also undertake the rescue obligation, even in the absence of explicit provisions in existing international conventions and related laws. This is because the home port, as the main place of operation of an international cruise ship, has the closest connection by nature with the ship and therefore should provide assistance to the endangered ship under any circumstance.

#### 4.1.3. The Right of Refusal to Entry

The rescue obligations of coastal and port states have limitations. Under the UNCLOS, the rescue obligation primarily concerns dangerous situations such as typhoons and collisions at sea, under which circumstances the nearby ships are obliged to render assistance when a cruise ship calls for rescue in international waters. The COVID-19 pandemic, whereas, is somewhat different from those circumstances. When a pandemic occurs, not only does the cruise ship require special treatment, but the rescuers, including the port states, also need to consider whether they have the necessary capability to prevent and control infectious disease. This is particularly the case in the early stages of the COVID-19 outbreak when many things were unknown in terms of transmission, containment, and treatment. It would be unjustified to request a costal or port state to undertake rescue obligations while putting its own people at risk. Therefore, in the case of the Westerdam cruise ship, Guam refused to rescue by stating that it had limited resources to “screen, quarantine, or treat 1400 patients at one time”, and its “obligation [was] to protect the people of Guam” first [20]. Other ports in Japan, South Korea, Thailand, and the Philippines also denied the ship from entry for similar reasons.

Under UNCLOS, innocent passage through territorial sea is an important right of all ships, as long as it is not prejudicial to the peace, good order, or security of the coastal state [21]. As a balance, the coastal state may take necessary steps in its territorial sea to prevent passage that is not innocent and, in its contiguous zone, may also exercise control necessary to prevent infringement of its custom, immigration or sanitary laws within its territorial sea [22]. Accordingly, the premise of an international cruise ship’s innocent passage is not to jeopardize the security of coastal states. In other words, if the coastal state deems that the infected international cruise ship may pose a threat to its own safety, it may choose to deny entry of the ship.

If a ship may bring about serious safety threats, costal states are inclined to refuse its entry. Environmental pollution excuses are mostly seen in the past. For example, in November 2002, when the Greek-operated oil tanker MV Prestige carrying 77,000 tonnes of heavy fuel oil was in danger while passing through the waters near Spain, not only the Spanish but the French and Portuguese governments also refused to allow the ship to dock, as these costal states claimed that such an oil leakage accident would cause tremendous damage to their local ecological environment. Compared with oil pollution, the impact of an unpredictable pandemic could be greater. Hence, it can be assumed that the coastal and port states were under pressure to deny entry of an infected international cruise ship in the COVID-19 pandemic circumstance.

### 4.2. Who Is Accountable?

Apart from the coastal and port states, there are other stakeholders who are also accountable for the prevention and control of COVID-19 on international cruise ships, we found that the flag state, the cruise ship company, and the states of the nationalities of onboard passengers and crew members are also legally involved.

#### 4.2.1. Flag State

The flag state of an international cruise ship is the jurisdiction under whose laws the ship is registered or licensed, namely, the nationality of the ship. Many international agreements including various IMO conventions require the flag states to effectively exercise their jurisdiction and control in administrative, technical and social matters over ships flying their flags [23]. Hence, when encountering COVID-19 issues, a cruise ship can choose to call at the port of its flag state. Even if the ship is in the waters of other states, its flag state cannot be exempted from those obligations and responsibilities under the flag state principle.

The UNCLOS requires that all ships should have the nationality of the state whose flag they are entitled to fly, and there must be a genuine link between the real owner of the ship and the flag with which the ship flies [24]. However, for various reasons and for a long time, to date there is still no uniform standard in implementing this crucial rule, which has led to the so-called “flag of convenience” problem. According to the UNCTAD’s latest Review of Maritime Transport, in 2019, 7 of the world’s top 10 ship-owning states had their national flags weight under 20% [25]. Under COVID-19, even though they are legally bound, flag states have neither pressure nor motivation to undertake epidemic prevention and control responsibilities. This is also the underlying reason why most flag states chose to shy away from exercising their jurisdictions when their cruise ships encountered COVID-19 problems.

#### 4.2.2. Cruise Ship Company

While a flag state has the legal authority and responsibility to enforce inspection and safety regulations on international cruise ships that are registered under its flag, the safe operation of ships and the safety of people onboard are primarily the ship operators’ responsibility. Article 1 of the IHR defines an operator as “a natural or legal person in charge of a conveyance or their agent,” which may include ship operators, ship managers and other enterprises, generally referred to as cruise ship companies. Under the current international law framework, there are regulations that specify responsibilities of the states and cruise ship companies with respect to safety and health issues. Article 24 of the IHR, in particular, provides that a state shall take all practicable measures to ensure that ship operators (a) comply with the health measures recommended by WHO and adopted by the state, (b) inform travelers of the health measures recommended by WHO and adopted by the state for application on board, and (c) permanently keep ships free of sources of infection or contamination, including vectors and reservoirs. Corresponding measures to control sources of infection or contamination may be required to apply if evidence is found. When the COVID-19 pandemic is confirmed as a PHEIC, cruise ship companies are obligated to further strengthen their healthcare measures and to cooperate with authorities of coastal and port states as well as their flag state so as to carry out epidemic prevention and control actions effectively, which includes reporting the ship’s actual situation quickly and accurately.

#### 4.2.3. State of Nationality of the People on Board

As far as the protection of human rights is concerned, the states of nationality of passengers and crew members on international cruise ships are also legally accountable when they are in danger during the COVID-19 pandemic. Both international treaties, such as the International Covenant on Economic, Social and Cultural Rights (ICESCR), and national laws contain relevant provisions that require the state of the nationalities of the people on board to protect its citizens, which entails protecting the right to health, i.e., “the highest attainable standards of physical and mental health” [26]. From this perspective, all involved states of nationality are obligated to rescue the infected cruise ship in order to protect their nationals’ basic right to health. In the Diamond Princess case, largely out of humanitarianism considerations, Japan eventually decided to permit onboard people to disembark so as to take care of their health. Other states of nationality in this case, such as China and the United States, also arranged to evacuate their nationals and to quarantine them further in their own countries. Indeed, as a UN expert recently pointed out, in the fight against the unprecedented COVID-19 pandemic, binding obligations shall be grounded first and foremost on the right to health framework which compels all parties involved to examine the adequacy of their measures [27].

### 4.3. Rationality and Legality of Certain Measures

#### 4.3.1. Quarantine at Sea

While the Diamond Princess cruise ship was at berth in the Yokohama Port, the Japanese authorities decided to quarantine the entire ship, including all passengers and crew members, on the sea instead of at a designated medical institution on shore for inspection and observation. Some of the measures brought about challenges in terms of rationality and legality.

According to related international agreements, such as the Convention on the International Regime of Maritimes Ports, the assessing criteria mainly include the necessity, reasonableness, appropriateness, and non-discrimination of applying the measures. Article 28(5) of the IHR provides that, if a suspect or affected ship berths elsewhere than at the port at which the ship was due to berth for reasons beyond the control of the officer in command of the ship, as soon as the competent authority has been informed, it may apply health measures recommended by the WHO or other IHR health measures. Nevertheless, these criteria are generally broad, and, apart from them, there are no other compulsory requirements under existing international legal frameworks. Hence, whether a state’s specific measures are legitimate or not, it should be assessed by the applicable laws of the concerned state.

When the Diamond Princess was permitted to berth, Japan as the port state had the discretionary right to take inspection and sanitary measures deemed necessary in accordance with its own domestic laws so as to prevent and control the pandemic, including requiring mandatory quarantine for 14 days. However, the consequence of applying these measures during the quarantine period was that the number of confirmed COVID-19 cases on board continued to rise. There are reasons to doubt that such a situation is directly linked with the quarantine environment of the ship itself as well as certain measures adopted by the Japanese authority. In other words, it appeared that some of Japan’s measures failed to fully consider or even ignored whether the cruise ship itself has the necessary conditions for an effective quarantine. If this can be established, then the rationality and appropriateness of these measures adopted on board would be subject to challenge.

#### 4.3.2. Prohibiting Persons on Board from Disembarking

Another controversial measure taken by the Japanese government in the Diamond Princess case was that all passengers and crew members on board were prohibited from disembarking, while some held that those healthy people should be allowed to go on shore first. Article 28(2) of the IHR stipulates that ships “shall not be prevented from embarking or disembarking” for public health reasons. However, again, this may be subject to inspection or other measures necessary to prevent the spread of infection or contamination. Article 43(1) further provides that in response to specific public health risks or PHEIC, a country may apply additional health measures in accordance with its relevant national laws if these measures can achieve the same or a greater level of health protection than WHO recommendations. Such measures shall not be more restrictive of international traffic and not more invasive or intrusive to persons than reasonably available alternatives that would achieve the appropriate level of health protection. In addition, from a human rights perspective, IHR Article 3 requires that “the implementation of these Regulations shall be with full respect for the dignity, human rights and fundamental freedoms of persons”. As such, if a country adopts certain public health measures that impose restrictions on free movement or require other interventions at a personal or community level, these measures must consist with human right protection requirements and be balanced with ethical considerations.

In the case of the Diamond Princess, because of the poor circulation of fresh air on board, especially in narrow cabins, being quarantined in such a confined space would be more likely to increase cross-infection. Even for healthy people, their immunity would decrease as a consequence of excessive psychological pressure who suffered from the depressive environment, therefore increasing the probability of infection [28]. In other words, those healthy people quarantined on board are below the appropriate level of health protection and would be more likely to be infected than under normal circumstances. If fact, on the Diamond Princess, it was reported that quarantined passengers and crew members increasingly felt helpless, anxious, and fearful, and over time various degrees of mental and physical exhaustion were common [29]. There are studies showing that close confinement helps the coronavirus to spread on board, and passengers could still infect their room-mates and crew members during cabin quarantine [30]. In this sense, Japan’s prohibition of healthy people onboard from disembarking appears problematic.

## 5. Conclusions

During the COVID-19 pandemic, many international cruise lines were suspended in an effort to prevent the global spread of the new coronavirus disease. Some national governments prohibited foreign cruise ships from entering their ports, making thousands of passengers and seafarers unable to disembark while the ships were at sea. These predicaments highlight the lack of international cooperation and coordination for handling such emergencies. COVID-19 is not the first time that international cruise ships encountered predicaments because of a pandemic. After establishing the public health emergency mechanism under the IHR, the WHO has declared the PHEIC six times. SARS in 2003, the H1N1 influenza in 2013, the Ebola virus disease in 2014, and MERS in 2015 affected the global cruising industry substantially. Similar public health problems are likely to happen again in the future. Therefore, we make the following recommendations for the prevention and control of highly contagious epidemics such as COVID-19 on international cruise ships.

First, it must be recognized that in the era of globalization and in the face of a global pandemic, no single state can manage everything, nor can any single international organization solve the problems independently [31]. The predicaments international cruise ships encountered in the early stages of the COVID-19 pandemic, including the above-mentioned legal controversies, clearly indicate that international coordination and cooperation in this field must be strengthened and improved systemically. For instance, the rapid spread of coronavirus on some cruise ships partially resulted from the design of ships, which prevents the effective inspection and isolation of the disease, and a shortage of quarantine and medical facilities, screening, and monitoring protocols. Therefore, in terms of the technical standards of cruise ships, the international community should jointly revise and upgrade the construction specifications and epidemic prevention standards of cruise ships by means of, inter alia, coordinating expert groups on a ship’s air-conditioning systems, ship design, operation, and management, and promoting these more advanced technical standards to key stakeholders via international organizations such as the International Association of Classification Societies (IACS). In the meanwhile, it must be acknowledged that the capabilities of different stakeholders are varied and a national government’s priority is always to protect its own citizen’s rights. Any future international cooperation shall take into consideration the imbalances among all stakeholders.

Information sharing and management is also crucial for the prevention and control of the pandemic. Operating international cruise lines usually involves multiple ports, states, and regions; in the case of epidemic outbreaks, cruise ship companies are expected to work closely with relevant public health authorities to enforce health requirements. It is particularly necessary for them to collect information on the occurrence and development of the epidemic quickly and accurately and to establish a risk assessment mechanism so as to cope with public health emergencies. In this regard, we advise the port states to consider epidemics as an essential indicator in assessing the safety of cruise ships, make files for each visiting ship, and review their emergency response mechanisms regularly.

All this international cooperation should be rule-based and under the framework of relevant international organizations including the WHO, the IMO, the ILO, and the International Chamber of Shipping (ICS). Their primary mission is to coordinate diversified regulations and practices of different countries, make suggestions on establishing harmonized or even unified rules, and evaluate whether a country is fulfilling its responsibilities and obligations under relevant legal frameworks. In view of the regulatory controversies that occurred during the COVID-19 pandemic, these international organizations are particularly expected to take a leading role in evaluating key international rules embodied in the IHR, the International Labour Convention, the Convention on Facilitation of International Maritime Traffic and the International Convention for the Safety of Life at Sea. We recommend that these organizations be more actively involved in addressing those new legal issues under the COVID-19 pandemic circumstances by way of improving existing cooperation and surveillance mechanisms of involved parties. In view of strengthening the future global health governance framework, a more effective supervisory mechanism is highly recommended so as to harmonize various national laws on pandemic control with the international health standards.

With respect to the specific rules in relation to the prevention and control of COVID-19 on international cruise ships, the future legal frameworks are expected to define more clearly the roles and responsibilities of all stakeholders, especially the coastal states, port authorities, and cruise operators. As a general rule, human rights protection shall be the priority consideration when humanitarian crises occur due to the quarantine of people onboard. Contemporary international law has actually been increasingly highlighting human rights, such as the rights “to just and favourable conditions of work [and] to rest and leisure, including reasonable limitation of working hours and periodic holidays with pay” as stipulated by the UN Human Rights Charter [32]. The general duties to render assistance under the UNCLOS, including requiring coastal states to rescue people whose safety are endangered at sea, are also for humanitarian protection [33]. During the COVID-19 pandemic in 2020, numerous seafarers were stranded on cruise ships and were unable to return home as a result of travel restrictions imposed by various states, leading to a humanitarian and safety crisis [34]. Their fundamental human rights were apparently not well respected and protected. An encouraging move was that, on 8 December 2020, the ILO called for urgent action by adopting a special resolution to address the situation and placed the issue of seafarer’s rights at the forefront of state consideration [35]. This is the direction in which the international community shall direct their efforts, and a more explicit highlighting of applicable human rights regulations shall be included in future international health law frameworks.

**Figure 1:**
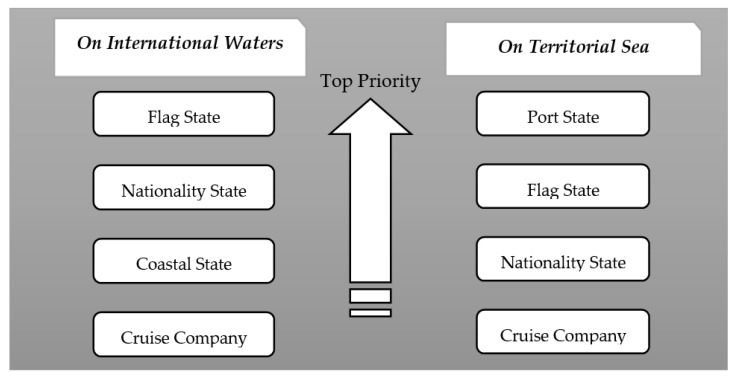
Priority Order of Stakeholders undertaking Responsibilities.

In line with the human rights principle as well as other UNCLOS rules such as the flag state principle, we recommend further clarification on exercising jurisdiction and distributing responsibilities, so that all involved parties can provide the necessary assistance as much and as conveniently as possible in response to COVID-19 control on international cruise ships. Figure 1 presents the priority order of stakeholders undertaking responsi-bilities. More specifically, when a cruise ship is sailing on international waters that are not within any of the state’s jurisdiction, the flag state shall maintain its prior right and obligation to rescue the ship. Other coastal states and states of nationality may exercise jurisdiction on the basis of human rights protection. However, when the ship is on the territorial sea of a coastal state and called at a specific port, we recommend that the port state take precedence so as to implement public health measures in accordance with the IHR and other applicable laws. In the meantime, all other stakeholders of the infected ship, including its flag state, the states of the nationality of those on board, and cruise ship companies, shall also actively coordinate and cooperate to fulfill their responsibilities, respectively. That is, a multiple or combined responsibility mechanism is highly recommended.

## Figures and Tables

**Table 1:** List of selected COVID-19 cases on cruise ships.

Ship Name	Passenger	Crew	Cases	Dock/Location	Owner/Operator
Artania	800	500	89	Fremantle, Australia	Phoenix Reisen, German
Braemar	682	38	5	Mariel, Cuba	FOCL, Norway
Coral Princess	1020	878	12	Miami, USA	Princess Cruises, Bermuda
Costa Luminosa	1370	410	36	Marseille-Fos, France	Costa Cruises, Italy
Costa Magica	2309	945	2	Miami, USA	Costa Cruises, Italy
Diamond Princess	2666	1045	712	Yokohama, Japan	Princess Cruises, Bermuda
Grand Princess	2422	1111	122	Oakland, USA	Princess Cruises, Bermuda
Paul Gauguin	148	192	1	Papeete, France	Ponant, France
River Anuket	101	70	45	Luxor, Egypt	Holland America, USA
Roald Amundsen	177	160	36	Tromsø, Norway	Hurtigruten, Norway
Silver Shadow	318	291	2	Recife, Brazil	Royal Caribbean, USA
Westerdam	781	747	1	Sihanoukville, Cambodia	Holland America, USA
World Dream	1871	1820	12	Hong Kong, China	Dream Cruises, China
Zaandam	1243	586	11	Everglades, USA	Holland America, USA

## Data Availability

The data presented in this study are available on request from the corresponding author.
